# Quinolone resistant *Salmonella* species isolated from pediatric patients with diarrhea in central Iran

**DOI:** 10.1186/s12876-021-01719-3

**Published:** 2021-03-30

**Authors:** Elnaz Abbasi, Ehsanollah Ghaznavi-Rad

**Affiliations:** 1 Department of Microbiology and Immunology, Faculty of Medicine, Arak University of Medical Sciences, Arak, Islamic Republic of Iran; 2 Molecular and Medicine Research Center, Faculty of Medicine, Arak University of Medical Sciences, Arak, Islamic Republic of Iran; 3 Department of Medical Laboratory Sciences, School of Allied Medical Sciences, Arak University of Medical Sciences, Arak, Iran

**Keywords:** *Salmonella* spp., Diarrhea, Antibiotic, Quinolone resistance, Iran

## Abstract

**Background:**

This study aimed to investigate the frequency and the antibiotic resistance patterns of *Salmonella* species that were isolated from infectious diarrhea samples taken from pediatric patients in central Iran.

**Methods:**

The study analyzed 230 stool specimens that were cultured on XLD, MacConkey agar and GN broth. Polymerase chain reaction (PCR) assay was used to identify the *Salmonella* genus. The antibiotic resistance profiles and the frequency of quinolone and integron genes were obtained.

**Results:**

Out of 230 samples of infectious diarrhea, 21 (9.1%) cases of *Salmonella* spp. were identified using culture methods. Another 28 (12.1%) samples had positive PCR results, with *S.* serovar Paratyphi B and C (9/21; 42.8%) and *S.* Typhi (3/21; 14.3%) being the most recognized. The highest antibiotic resistance rates were found for nalidixic acid (15/21; 71.4%), tetracycline (9/21; 42.8%). However, six (28.5%) of isolates were found resistant to cotrimoxazole, ampicillin and chloramphenicol. Among the plasmid-mediated quinolone resistance (PMQR) determinants, *qnrS*, *qnrA*, and *qnrB* were positive in (9/15; 60%), (6/15; 40%) and (3/15; 20%) of the isolates, respectively. Class 1 and 2 integrons were identified in 15 (71.4%) and 3 (14.3%) isolates, respectively.

**Conclusion:**

High rates of quinolone resistant and low frequency of MDR *Salmonella* spp. isolates were identified in central Iran, similar to findings in other parts of Asia. To prevent the spread of these resistant strains, the antimicrobial resistance of *Salmonella* spp. isolates should be under constant surveillance, and empiric antibiotic therapy should be adapted appropriately.

## Background

Gastroenteritis remains a serious public health problem. This common human disease is the second leading cause of morbidity and mortality in developing countries, including Iran, and has a particularly high morbidity in children younger than 5 years old (with an estimated 525,000 deaths per year worldwide) [1]. Diarrheal diseases can negatively affect early pediatric growth both through enteric dysfunction and the impaired uptake of macronutrients and micronutrients [2]. *Salmonella* is a common cause of infectious gastroenteritis among the pediatric age group in many developing countries [3]. Globally, human salmonellosis causes 200 million to over 1 billion disease cases annually, with over 150 thousand of these cases resulting in death [4]. The *S. enterica* serovars Typhi (*S*. Typhi) and Paratyphi (*S*. Paratyphi A, B and C) are the most common causes of human salmonellosis worldwide [5]. Initially, the antimicrobial resistance profiles of these *Salmonella* serovars were determined against the antibiotics of ampicillin, co-trimoxazole (trimethoprim-sulfamethoxazole) and chloramphenicol, and bacteria with simultaneous resistance to all three types of antibiotics were characterized as multi-drug resistant (MDR) [6]. Currently, MDR typhoid is in decline in the Asian regions, where there is a high level of resistance to second-line drugs, such as quinolones and fluoroquinolones [6]. This increased antimicrobial resistance has decreased the number of effective treatment options and, consequently, increased the treatment costs, the risk of complications and the death rate, especially in the pediatric age group [3].

In the summer, diarrhea and dysentery are so common among the pediatric patients at central Iran [7–11]. It is not efficient to identify typhoidal *Salmonella* in clinical detection labs, and specific information about the scale of typhoidal *Salmonella* in Iran’s central region is not available. Therefore, this study has been conducted to examine in depth the abundance, the phenotypic antimicrobial resistance levels and the resistance gene content of the region’s *Salmonella* species by examining diarrhea samples from patients.

## Materials and methods

### Sample collection

This study protocol was approved by the ethics committee of the Arak University of Medical Sciences (ARAKMU.REC. 93-176-30 and 1395.83). All methods were performed in accordance with the relevant guidelines and regulations. For this cross-sectional, descriptive study, 230 samples of diarrhea were gathered from pediatric patients who were referred to the Children’s Educational-Therapeutic Center affiliated with Arak University of Medical Sciences (in the city of Arak, Iran) due to diarrhea from May 2015 to May 2016. The parent/guardian consent form was provided for participants under 16 years old.

The inclusion criteria for this study were as follows: a completed consent form and a questionnaire was filled out by the patient or the patient’s parents and caregivers; observation of more than five white blood cells per high-power field (HPF) in a stool specimen [12] and the patient had not taken antibiotics for a week before consultation at the hospital.

### Phenotypic investigation

The fecal samples were cultured in Gram-negative (GN) broth, xylose lysine deoxycholate (XLD) and MacConkey media (Merck, Hamburg, Germany); then biochemical and serological tests were performed [7]. Application programming interface (API) testing (Biomeriux, France) was used to confirm the presence of *Salmonella* spp. isolated. *S. enterica* subsp. enterica PTCC 1709, *S. enterica* subsp. enterica serovar Paratyphi A PTCC 1230, *S. enterica* subsp. enterica serovar Paratyphi B PTCC 1231 and *S.* Typhi PTCC 1609 were used as controls in each assay (obtained from the Iranian Research Organization for Science and Technology). *S. enterica* subsp. enterica serovar Paratyphi C control strains were acquired from the microbiology department of the Arak University of Medical Sciences.

### Investigating *Salmonella* antibiotic resistance by disk diffusion

Using the Clinical and Laboratory Standards Institute (CLSI) 2017 guidelines [13], an antibiogram assay was performed on the isolated *Salmonella* spp. colonies. The antibiotic discs contained nalidixic acid (30 μg), tetracycline (30 μg), cotrimoxazole (25 μg), ampicillin (10 μg), chloramphenicol (30 μg), cefixime (5 μg), ceftriaxone (30 μg), cefotaxime (30 μg), ceftizoxime (30 μg), ceftazidime (30 μg), cefoxitin (30 μg), cefepime (30 μg), gentamicin (10 μg), azithromycin (15 μg), ciprofloxacin (5 μg) and imipenem (10 μg) (Mast Diagnostics, United Kingdom).

### Genotypic investigations

#### DNA extraction

DNA was directly extracted from the fecal samples and the reference *Salmonella* spp. isolates using the QIAamp DNA stool mini kit (Qiagen GmbH, Hilden, Germany), according to the manufacturer’s protocol. The amount and purity of the extracted DNA were measured with a NanoDrop apparatus (Thermo Fisher Scientific, Waltham. Massachusetts, United States) and confirmed using the universal primers for the bacterial *16S* rRNA gene [8].

### Genotypic identification

PCR of the *inlA* gene was performed to confirm the *Salmonella* genus [14]. PCR of the *qnr* determinant genes *qnrS*, *qnrA*, and *qnrB* was performed to amplify the plasmid-mediated quinolone resistance (PMQR) targets. Mutations in the *gyrA* and *parC* genes of the quinolone-resistant *Salmonella* spp. isolates were also identified using DNA sequencing techniques [7]. *Sul*1,2 for sulfonamide resistance and quartenary ammonium compounds (*qac*) resistance genes were investigated using PCR method (Table 1) [15].

**Table 1:** The primers used in this study

Target gene description	Primer	Sequence 5′ → 3'	Amplicon size (bp)	Annealing temperature	References
Universal DNA bacterial	*16s-rRNA*-F*16s-rRNA*-R	5-AGGAGGTGATCCAACCGCA-35-ACCTGGAGGAAGGTGGGGAT-3	367	55	[8]
Salmonella spp.	*invA*-F*invA*-R	5- TTGTTACGGCTATTTTGACCA-35- CTGACTGCTACCTTGCTGATG-3	521	60	[14]
Fluoroquinolone	*gyrA*-F*gyrA*-R	5-AAATCTGCCCGTGTCGTTGGT-35-GCCATACCTACGGCGATACC -3	344	55	[7]
	*parC*-F*parC*-R	5-CTGAATGCCAGCGCCAAATT-35-GCGAACGATTTCGGATCGTC-3	168	55	[7]
	*qnrS*-F*qnrS*-R	5-TGGAAACCTACAATCATACATATCG-3 5-TTAGTCAGGATAAACAACAATACCC-3	656	60	[7]
	*qnrA*-F*qnrA*-R	5-GATAAAGTTTTTCAGCAAGAGG-35-ATCCAGATCGGCAAAGGTTA-3	593	60	[7]
	*qnrB*-F*qnrB*-R	5-GTTGGCGAAAAAATTGACAGAA-35-ACTCCGAATTGGTCAGATCG-3	264	53	[7]
Integrase1	*Int1*-F*Int1*-R	5-CAGTGGACATAAGCCTGTTC-35-CCCGAGGCATAGACTGTA-3	160	55	[7]
Integrase2	*Int2*-F*Int2*-R	5-TTGCGAGTATCCATAACCTG-35-TTACCTGCACTGGATTAAGC-3	288	55	[7]
Integrase3	*Int3*-F*Int3*-R	5-GCCTCCGGCAGCGACTTTCAG-35-ACGGATCTGCCAAACCTGACT-3	979	59	[7]
Sulfonamide resistance	*Sul1*-F*Sul1*-R	5-TCACCGAGGACTCCTTCTTC-35-CAGTCCGCCTCAGCAATATC-3	331	65	[7]
	*Sul2*-F*Sul2*-R	5-CCTGTTTCGTCCGACACAGA-35-GAAGCGCAGCCGCAATTCAT-3	435	58	[7]
Quaternary ammonium compounds	*qac*-F*qac*-R	5-GCCCTACACAAATTGGGAGA-35-CTGCGGTACCACTGCCACAA-3	370	55	[15]

### Integron detection

To investigate class 1, 2 and 3 integrons, PCR assay was performed as previously described in the literature (Table 1) [7].

## Results

Of the 230 analyzed samples, 21 (9.1%) and 28 (12.1%) were found to be positive for *Salmonella* spp. using the exclusive culture and PCR methods, respectively. All the culture-positive samples were identified as positive using PCR; and seven of the samples that were culture-negative were also identified as positive using PCR. Of the 21 patients (9.1%) afflicted with *Salmonella* spp., 9 (42.8%) were female and 12 (57.1%) were male, resulting in a female-to-male infection ratio of 1:1.3. The average age of the people afflicted with salmonellosis was 4 years and 5 months. The youngest diseased person was an 8-month-old girl; the oldest was a 12-year-old boy. The clinical symptoms among the people suffering from salmonellosis are given in Table 2.

**Table 2:** Frequency of clinical symptoms in pediatric patients with *Salmonella* spp.

*Salmonella* spp.	Mucus in the stool	Abdominal pain	Vomiting	Fever	Blood in the stool
*S*. Paratyphi B	9/9 (100%)	9/9 (100%)	6/9 (66.6%)	5/9 (55.5%)	6/9 (66.6%)
*S*. Paratyphi C	9/9 (100%)	7/9 (77.7%)	3/9 (33.3%)	8/9 (88.8%)	8/9 (88.8%)
*S*. Typhi	3/3 (100%)	3/3 (100%)	1/3 (33.3%)	3/3 (100%)	3/3 (100%)
*S*. Paratyphi A	–	–	–	–	–

### Phenotypic and genotypic investigation

Of the 21 cultured *Salmonella* spp. isolates, 9 (42.8%) were identified as *S*. Paratyphi B, 9 (42.8%) were identified as *S*. Paratyphi C, and 3 (14.3%) was identified as *S*. Typhi; no case of *S*. Paratyphi A was found.

### Phenotypic and genotypic antibiotic resistance determination

Using the CLSI 2017 guidelines, the highest resistance rates in *Salmonella* spp. were observed against nalidixic acid (15/21; 71.4%), tetracycline (9/21; 42.8%), cotrimoxazole (6/21; 28.5%), ampicillin (6/21; 28.5%), and chloramphenicol (6/21; 28.5%) (Table 3). All of the *Salmonella* isolates were susceptible to cefixime, ceftriaxone, cefotaxime, ceftizoxime, ceftazidime, cefoxitin, cefepime, gentamicin, azithromycin, ciprofloxacin, and imipenem. No cases of MDR were observed. All isolates carrying PMQR contain similar mutations in *parC* at amino acid 80 (replacement of serine with isoleucine; GenBank accession no. HM068910) and *gyrA* at amino acid 83 (replacement of serine with leucine). The frequency of antibiotic resistance genes among *Salmonella* spp. was given in Table 4.

**Table 3:** Phenotypic antibiotic resistance rates in *Salmonella* spp.

Antibiotic	*Salmonella* spp. n:21	*S*. Paratyphi B n:9	*S*. Paratyphi C n:9	*S*. Typhi n:3
Nalidixic acid	15 (71.4%)	6 (66.6%)	6 (66.6%)	3 (100%)
Tetracycline	9 (42.8%)	5 (55.5%)	2 (22.2%)	2 (66.6%)
Cotrimoxazole	6 (28.5%)	2 (22.2%)	3 (33.3%)	1 (33.3%)
Ampicillin	6 (28.5%)	4 (44.4%)	1 (11.1%)	1 (33.3%)
Chloramphenicol	6 (28.5%)	3 (33.3%)	2 (22.2%)	1 (33.3%)

**Table 4:** The frequency of antibiotic resistance genes among *Salmonella* spp.

Resistance	Target gene	*Salmonella* spp.	*S*. Paratyphi B	*S*. Paratyphi C	*S*. Typhi
Sulfonamide	*Sul1*	6/6 (100%)	2/2 (100%)	3/3 (100%)	1/1 (100%)
	*Sul2*	3/6 (50%)	2/2 (100%)	1/3 (33.3%)	0%
Fluoroquinolone	*gyrA*	15/15 (100%)	6/6 (100%)	6/6 (100%)	3/3 (100%)
	*parC*	15/15 (100%)	6/6 (100%)	6/6 (100%)	3/3 (100%)
	*qnrS*	9/15 (60%)	4/6 (66.6%)	2/6 (33.3%)	3/3 (100%)
	*qnrA*	6/15 (40%)	2/6 (33.3%)	1/6 (16.6%)	3/3 (100%)
	*qnrB*	3/15 (20%)	2/6 (33.3%)	0%	1/3 (33.3%)
Integrase	*Int1*	15/21 (71.4%)	7/9 (77.7%)	6/9 (66.6%)	2/3 (66.6%)
	*Int2*	3/21 (14.3%)	3/9 (33.3%)	0%	0%
	*Int3*	0%	0%	0%	0%
Quaternary ammonium compounds	*qac*	9/21 (42.8%)	5/9 (55.5%)	3/9 (33.3%)	1/3 (33.3%)

## Discussion

The frequency of salmonellosis, as determined by bacterial culture and PCR, was 9.1% and 12.1%, respectively. Of these two methods, the sensitivity of the PCR method was higher [16, 17]. Other studies conducted in Sudan, Iran (Tehran), Iraq reported frequencies of 4%, 7%, and 14.8%, respectively [14, 18, 19]. These differences in the frequency of salmonellosis may be related to a variety of factors, including exposure to the natural reservoirs of *Salmonella* species in these geographical areas, differences in climate and many other environmental conditions, as well as age differences, and differences in the level of economic development, the level of individual hygiene, and contamination via food preparers who are chronic carriers of *Salmonella* [5].

In the present study, the most prevalent *Salmonella enterica* serovar isolates were *S*. Paratyphi B and *S*. Paratyphi C (42.8%). In other studies, *S*. Typhi and *S*. Paratyphi B were reported to be the predominant serogroup in Iran (Tehran) and Ethiopia, respectively [19, 20]. General hygiene, socioeconomic conditions, and ecological conditions affect the frequency of *Salmonella* spp. serogroups [4].

Because salmonellosis is spread by food, any information regarding the frequency and the antimicrobial resistance of the isolates is a public health concern [21]. The antibiotic resistance properties of *Salmonella* spp have been reported to vary and be regionally distinct [22]. The present study is the first to report on the frequency of salmonellosis and its associated resistance patterns for a panel of 16 antibiotics in central Iran.

The traditional first-line drugs used to treat *Salmonella* spp. are chloramphenicol, ampicillin, and cotrimoxazole [6]. In Iran (Tehran) and Mexico, 11% and 33% of the strains have been reported to be resistant to chloramphenicol, respectively [3, 23]. In Iran (Tehran), Mexico, and Pakistan 13.5%, 20%, and 66.1% of the strains were found to be resistant to ampicillin [23–25]. In Iran (Tehran), Mexico, and Pakistan 23%, 28.8%, and 66.5% of the strains were resistant to cotrimoxazole, respectively [23–25]. These differences indicate that resistance to *Salmonella* first-choice agents may be related to presentation from different sources [26]. In Iran (Tehran), *Sul1* was found to be resistant in 32% of the strains [3]. In India, *Sul1* and *Sul2* were found to be resistant in 100% and 77.7% of the strains, respectively [27]. The difference in frequency of sulfonamide-resistant in different regions mainly relates to different antibiotic usage patterns [5]. However, MDR *Salmonella* spp. are a worldwide concern but is not very common [6].

In Iran (Tehran), Malaysia, and Nigeria, 51.8%, 42%, and 65.9% of the strains were resistant to tetracycline, respectively [28–30].

Although quinolone/fluoroquinolones are intended to be appropriate drugs against resistant isolates, the enhancement in antimicrobial resistance is a burden in controlling infections caused by *Salmonella* spp. [31]. In the present study, 71.4% of the *Salmonella* strains were found to be resistant to nalidixic acid, and none of the strains was resistant to ciprofloxacin. In Iran (Tehran), Nigeria, and India, 66.6%, 59%, and 96% of the strains were resistant to nalidixic acid, respectively [32–34].

In this study, *qnrS*, *qnrA*, and *qnrB* were found at 60%, 40%, and 20%, respectively, in nalidixic acid-resistant *Salmonella* strains. In Iran (Tehran), *qnrS*, *qnrA*, and *qnrB* were found at 56.5%, 30.4%, and 1.1% in the strains, respectively [32]. In Brazil, *qnrS*, *qnrB*, and *qnrA* were found at 53.3%, 40%, and 0% in the strains, respectively [31], while in India, *qnrB* was at 70% and none of the strains showed resistance to *qnrA* and *qnrS* [35]. Quinolones, and especially fluoroquinolones, are widely used in poultry farms and in the treatment of companion animals in Iran, and this contributes to the risk of resistant zoonotic bacterial agents being spread via the food chain [36]. Generally, studies have determined a direct relationship between quinolone usage in poultry and the frequency of nalidixic acid-resistant *Salmonella* spp. isolates from humans [37]. Fluoroquinolone resistance is prevalent across Asia, in part because of the widespread consumption of this class of antimicrobials [6].

In the current study, *qac* were found at 42.8% in Salmonella isolates, while in Iran (Tehran) and Iraq, *qac* were found at 31% and 60% in the strains, respectively [3, 38].

In Iran (Tehran), *int1* (32%), *int2* (13%), and *int3* (0%) were found in the strains [3], while in Hong Kong, *int1* was found in 13% of the strains but none showed resistance to *int2* and *int3* [39]. Of the three categories of integrons pertinent to antimicrobial resistance, the class I integron is the most frequently obtained in Gram-negative bacteria [40]. The prevalence of integrons in the enterobacteriacae family has been varied and has played a significant role in the development of drug-resistant bacteria [41]. Thus, the high prevalence of antibiotic resistance probably relates to the high prevalence of class I and II integrons.

## Conclusion

To reduce and prevent outbreaks of quinolone resistance, and prevention of the emergence of MDR *Salmonella* spp., a coherent program needs to be developed for the control and surveillance of antimicrobial resistance in the long run. Further, empiric antibiotic therapy should be adapted appropriately, and *Salmonella* carriers should be identified and given specific treatment in order to prevent this transmission route.

## Data Availability

All data pertaining to this study are within the manuscript in sections sample collection and results. The datasets analyzed and/or used during the current study are available from the corresponding author on reasonable request.

## Abbreviations

PCR: Polymerase chain reaction
DNA: Deoxyribonucleic acid
XLD: Xylose lysine deoxycholate
PMQR: Plasmid-mediated quinolone resistance
MDR: Multi-drug resistant
HPF: High-power field
BLAST: Basic local alignment search tool
